# Blood immune indexes can predict lateral lymph node metastasis of thyroid papillary carcinoma

**DOI:** 10.3389/fendo.2022.995630

**Published:** 2022-08-31

**Authors:** Lingqian Zhao, Tianhan Zhou, Wenhao Zhang, Fan Wu, Kecheng Jiang, Bei Lin, Siqi Zhan, Tao Hu, Tian Tang, Yu Zhang, Dingcun Luo

**Affiliations:** ^1^ Zhejiang Chinese Medical University, Fourth Clinical Medical College, Hangzhou, China; ^2^ Hangzhou Traditional Chinese Medicine Hospital Affiliated to Zhejiang Chinese Medical University, The Department of General Surgery, Hangzhou, China; ^3^ Affiliated Hangzhou First People’s Hospital Zhejiang University School of Medicine, Department of Oncological Surgery, Hangzhou, China

**Keywords:** papillary thyroid carcinoma, lateral cervical lymph node, lymph node metastasis, systemic immune-inflammation index, nomogram

## Abstract

**Objective:**

To explore the clinical significance of blood immune indexes in predicting lateral lymph node metastasis (LLNM) of thyroid papillary carcinoma (PTC).

**Methods:**

The pathological data and preoperative blood samples of 713 patients that underwent thyroid surgery at affiliated Hangzhou First People’s Hospital Zhejiang University School of Medicine from January 2013 to June 2021 were collected as the model group. The pathological data and preoperative blood samples of 177 patients that underwent thyroid surgery in the same hospital from July 2021 to October 2021 were collected as the external validation group. Univariate and multivariate logistic regression analyses were used to determine the independent risk factors of LLNM in PTC patients. A predictive model for assessing LLNM in PTC patients was established and externally validated using the external data.

**Results:**

According to univariate and multivariate logistic regression analyses, tumor diameter (P < 0.001, odds ratios (OR): 1.205, 95% confidence interval (CI): 1.162–1.249) and the preoperative systemic immune-inflammation index (SII) (P = 0.032, OR: 1.001, 95% CI: 1.000–1.002) were independent risk factors for distinguishing LLNM in PTC patients. When the Youden index was the highest, the area under the curve (AUC) was 0.860 (P < 0.001, 95% CI: 0.821–0.898). The externally validated AUC was 0.827 (P < 0.001, 95% CI: 0.724–0.929), the specificity was 86.4%, and the sensitivity was 69.6%. The calibration curve and the decision curve indicated that the model had good diagnostic value.

**Conclusion:**

Blood immune indexes can reflect the occurrence of LLNM and the biological behavior of PTC. The predictive model established in combination with SII and tumor diameter can effectively predict the occurrence of LLNM in PTC patients.

## Introduction

According to the latest global cancer data released by the World Health Organization (GLOBOCAN 2018), in 2018, more than 567,000 new cases of thyroid cancer were diagnosed in 20 regions in the world, and the incidence of thyroid cancer in women was as high as 10.2/100,000, approximately three times that of men (1). Papillary thyroid carcinoma (PTC) is the most common type of thyroid cancer. Although the prognosis is good, 9.1–38% of patients with PTC develop lateral lymph node metastasis (LLNM), which seriously affects the prognosis (2). We found that 5.6% of PTC patients with tumor diameter <10 mm had lateral neck lymph node metastasis (3). A recent single-sequencing study has found that tumor ecosystems and immune microenvironments can affect the occurrence and development of PTC (4). Meanwhile, other studies have reported that preoperative blood immune indexes, such as the neutrophil/lymphocyte ratio (NLR), lymphocyte/monocyte ratio (LMR), platelet/lymphocyte ratio (PLR), and preoperative systemic immune-inflammatory index (SII), can be used to assess immune function and diagnose tumors, and all have a good effect on tumor prediction (5, 6). However, the correlation of LLNM in PTC with systemic immunity and blood immune indexes is not clear. The purpose of this study is to investigate the clinical significance of preoperative blood immune indexes on LLNM in PTC patients, as well as to establish and verify the clinical predictive model and to guide the diagnosis and treatment of metastatic PTC.

## Materials and methods

### General information

The relevant data of 713 patients that underwent thyroid surgery at Affiliated Hangzhou First People’s Hospital Zhejiang University School of Medicine (Hangzhou, China) from January 2013 to June 2021 were collected for retrospective analysis. The relevant data of 177 patients that underwent thyroid surgery at the same hospital from July 2021 to October 2021 were collected for external verification analysis. The inclusion criteria were as follows: (1) postoperative pathological diagnosis of PTC and (2) unilateral gland lobe plus isthmus or full gland lobectomy, coupled with dissection of the lateral cervical lymph nodes, with evidence of lymph node metastasis in the lateral cervical region. The exclusion criteria were as follows: (1) evidence of other malignancies, (2) evidence of blood system diseases, (3) evidence of autoimmune system diseases, (4) evidence of preoperative acute or chronic inflammation or other diseases that could affect routine blood tests, and (5) incomplete clinical information.

### Data collection

Fasting peripheral blood was collected from the patients in the morning one week before thyroid surgery and processed by the Mindary BC-6800 automatic blood cell analyzer (Shenzhen Mairui Biomedical Electronics Co., Ltd., Shenzhen, China) and supporting reagents. Peripheral blood cells were classified and counted by the sheath flow impedance method, laser light scattering method, and flow cytometry combined with fluorescent staining. The absolute value of neutrophils, absolute value of lymphocytes, absolute value of monocytes, and absolute value of platelets were obtained. The absolute value of neutrophils/absolute value of lymphocytes (NLR), absolute value of platelets/absolute value of lymphocytes (PLR), absolute value of lymphocytes/absolute value of monocytes (LMR), and absolute value of platelets × absolute value of neutrophils/absolute value of lymphocytes (SII) were calculated.

Color doppler ultrasound machines equipped with a real-time, high-frequency (5-10 MHZ) linear-array probes were used in this study. The maximum diameter of thyroid tumors was interpreted by two sonographers with 3-5 years of experience in ultrasonic diagnosis before surgery. If the results of the two patients were inconsistent, the third sonographer with 3-5 years of experience in ultrasonic diagnosis would interpret and compare the results to determine the final preoperative maximum diameter of the thyroid tumor.

The general information of the patients, including gender and age, and the tumor information, including evidence of unilateral or bilateral tumors, multifocality and evidence of postoperative pathology confirmed LLNM, were collected.

### Statistical methods

SPSS software (version 26; IBM, Armonk, NY, USA) was used for the statistical analysis of the data. For measurement data, a normality test was carried out, expressed as mean ± standard deviation (± S), and a t-test was used for comparisons between two independent groups. A nonparametric test was used for comparisons between groups. The count data were expressed as frequency and percentage, and comparisons between groups were carried out with the chi-square (X^2^) test. By the t-test and X^2^ test, the influencing factors showing statistically significant differences were taken as the risk factors related to LLNM in PTC patients.

Logistic multivariate regression analysis was used to analyze the risk factors identified by univariate regression analysis. Differences in the preoperative blood inflammatory indexes and pathological characteristics of LLNM were analyzed, and the independent risk factors affecting LLNM in PTC patients were identified. The odds ratios (OR) and 95% confidence intervals (95% CI) were determined.

According to the results of logistic multivariate regression analysis, a predictive model was constructed, and receiver operator characteristic (ROC) curve analysis was used to evaluate the area under the curve (AUC) and its 95% CI, calculate the Youden index (sensitivity + specificity -1), and determine the sensitivity and specificity when the Youden index was at its highest. The predictive model was constructed, and the model was validated by random sampling 1000 times using the bootstrap method. The models were fitted using the external validation data, and AUC, 95% CI, sensitivity, and specificity were assessed. Lastly, a calibration plot was constructed. The performance of the predictive model was evaluated by the Hosmer–Lemeshow test, AUC, and goodness-of-fit. Decision curve analysis (DCA) was used to validate the clinical net benefit rate of the predictive model. Statistical analysis was performed using R studio (version 4.1.0). P < 0.05 was considered statistically significant.

## Results

### General information

A total of 713 patients were enrolled in this study. According to the inclusion and exclusion criteria, 702 patients with PTC were enrolled in the final model group, including 173 males (24.6%) and 529 females (75.4%), with an average age of 46.4 years. In the model group, there were 106 patients (15.1%) with LLNM and 596 (84.9%) without LLNM. The external validation group was comprised of 171 patients, including 46 males (26.9%) and 125 females (73.1%), with an average age of 45.5 years. In the external validation group, there were 22 patients (12.9%) with LLNM and 149 (87.1%) without LLNM. The general characteristics of the patients are shown in **Table 1**.

**Table 1:** General information of papillary thyroid carcinoma patients in model and external validation groups.

		Model group (702)		External validation group (171)	
		Without LLNM	With LLNM	Without LLNM	With LLNM
Total		596	106	149	22
Sex	Male	139	34	34	12
	Female	457	72	115	10
Age	$\bar{X}$ ± S	47.2 ± 12.3	42.3 ± 14.4	46.1 ± 12.5	41.7 ± 14.2
Tumor diameter (mm)	$\bar{X}$ ± S	8.1 ± 4.8	19.4 ± 11.1	6.6 ± 4.7	13.0 ± 7.7
Multifocality	Yes	194	47	44	9
	No	402	59	105	13
Bilateral	Yes	131	38	20	7
	No	465	68	129	15
NLR	$\bar{X}$ ± S	1.96 ± 1.46	2.06 ± 0.88	1.81 ± 0.84	1.92 ± 1.02
PLR	$\bar{X}$ ± S	132.12 ± 45.9	137.44 ± 51.74	126.72 ± 36.76	128.94 ± 45.65
LMR	$\bar{X}$ ± S	5.89 ± 2.42	5.58 ± 2.68	5.45 ± 2.20	4.99 ± 1.79
SII	$\bar{X}$ ± S	429.43 ± 271.25	492.23 ± 233.51	406.58 ± 217.22	471.91 ± 288.17

### Screening of predictive model variables

Gender, age, tumor diameter, multifocality, bilateral tumor, NLR, PLR, LMR, and SII were included in the analyses that employed the t-test, nonparametric test, and X^2^ test. The results of univariate regression analysis showed that age, tumor diameter, SII, bilateral tumor, and multifocality were significantly associated with LLNM in PTC (P < 0.05). However, there were no significant differences between gender, PLR, NLR, LMR, and LLNM in PTC (**Table 2**).

**Table 2:** General information and results of univariate logistic regression analysis of 702 papillary thyroid carcinoma patients.

		Total	LLNM		P-value (univariate analysis)	P-value (logistic regression analysis)	OR (logistic regression analysis)	95%CI (logistic regression analysis)	Regression coefficients (logistic regression analysis)
			Yes	No					
Total		702	106	596					
Sex	Male	173	34	139	0.066				
	Female	529	72	457					
Age	$\bar{X}$ ± S	46.5 ± 12.7			0.001	0.147			
Tumor diameter (mm)	$\bar{X}$ ± S	9.8 ± 7.4			<0.001	<0.001	1.205	1.162–1.249	0.186
Multifocality	Yes	241	47	194	0.020	0.586			
	No	461	59	402					
Bilateral	Yes	169	38	131	0.003	0.677			
	No	533	68	465					
NLR	$\bar{X}$ ± S	1.97 ± 1.39			0.466				
PLR	$\bar{X}$ ± S	132.92 ± 46.87			0.277				
LMR	$\bar{X}$ ± S	5.84 ± 2.46			0.231				
SII	$\bar{X}$ ± S	438.92 ± 266.69			0.025	0.032	1.001	1.000-1.002	0.001

Age, tumor diameter, SII, bilateral tumor, and multifocality were included in logistic multivariate regression analysis. The results showed that tumor diameter (P < 0.001, OR: 1.205, 95% CI: 1.162–1.249) and SII (P = 0.032, OR: 1.001, 95% CI: 1.000–1.002) were independent risk factors for LLNM in PTC.

### Model establishment

**Figure 1:**
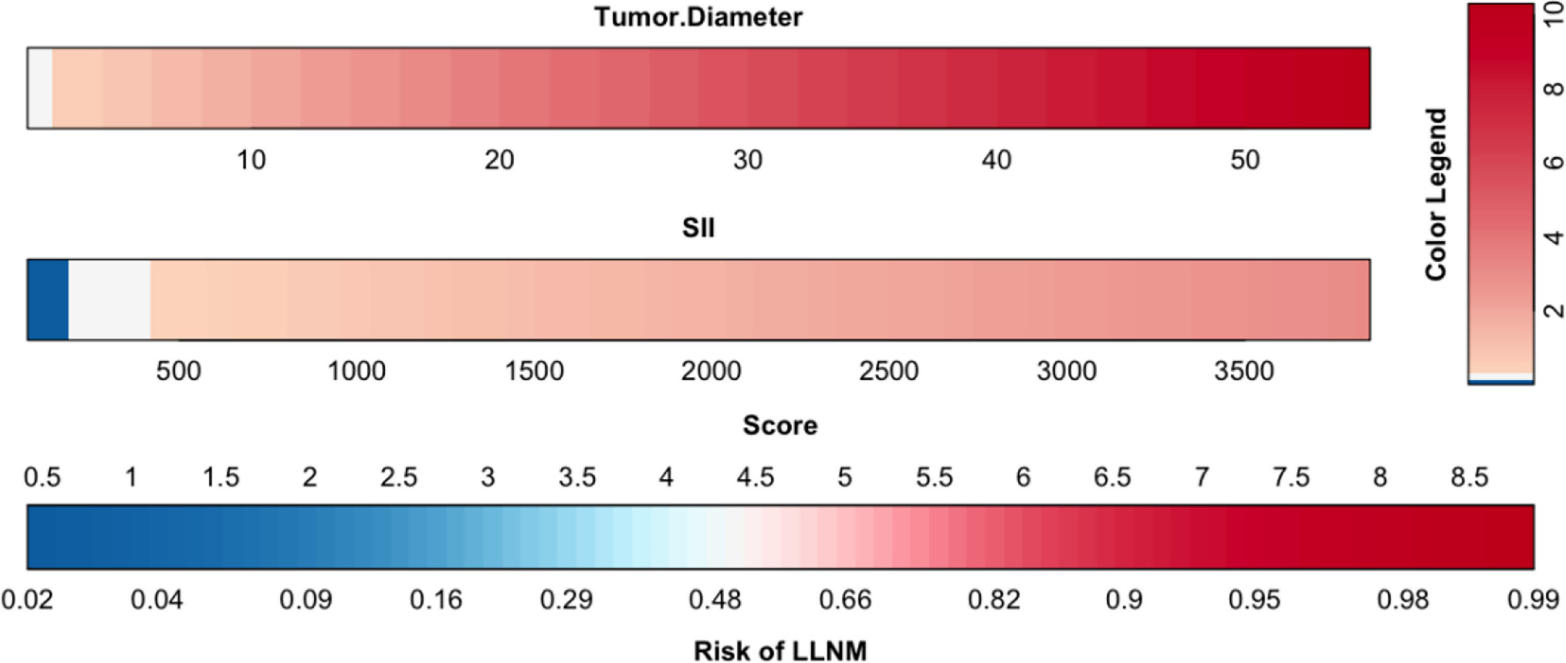
Clinical predictive model of lateral lymph node metastasis in papillary thyroid carcinoma patients.

**Figure 2:**
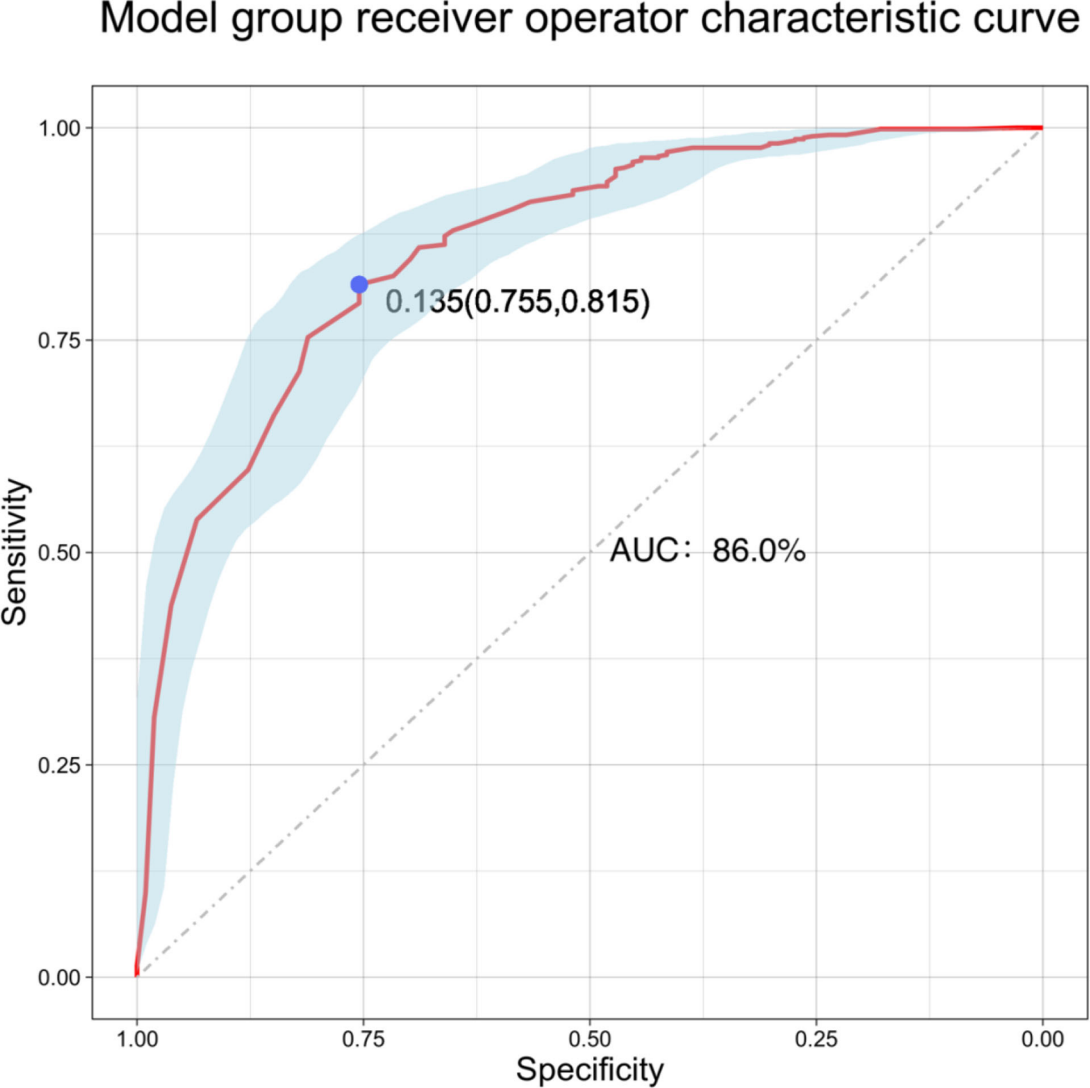
Model group receiver operator characteristic curve.

The results of logistic multivariate regression analysis were included in the construction of the predictive model, and the independent risk factors of LLNM in PTC included tumor diameter and SII. Thus, these independent predictors were combined to establish a predictive model to distinguish LLNM in PTC (**Figure 1**). The ROC curve was established according to the logistic multivariate regression results. The results showed that the AUC of the two risk factors, namely, tumor diameter and SII, for predicting LLNM in PTC patients was 0.860 (P < 0.001, 95% CI: 0.821–0.898). When the Youden index was the highest, the specificity was 75.5% and the sensitivity was 81.5%, which showed good discrimination (**Figure 2**).

### Model checking

#### Model internal inspection

**Figure 3:**
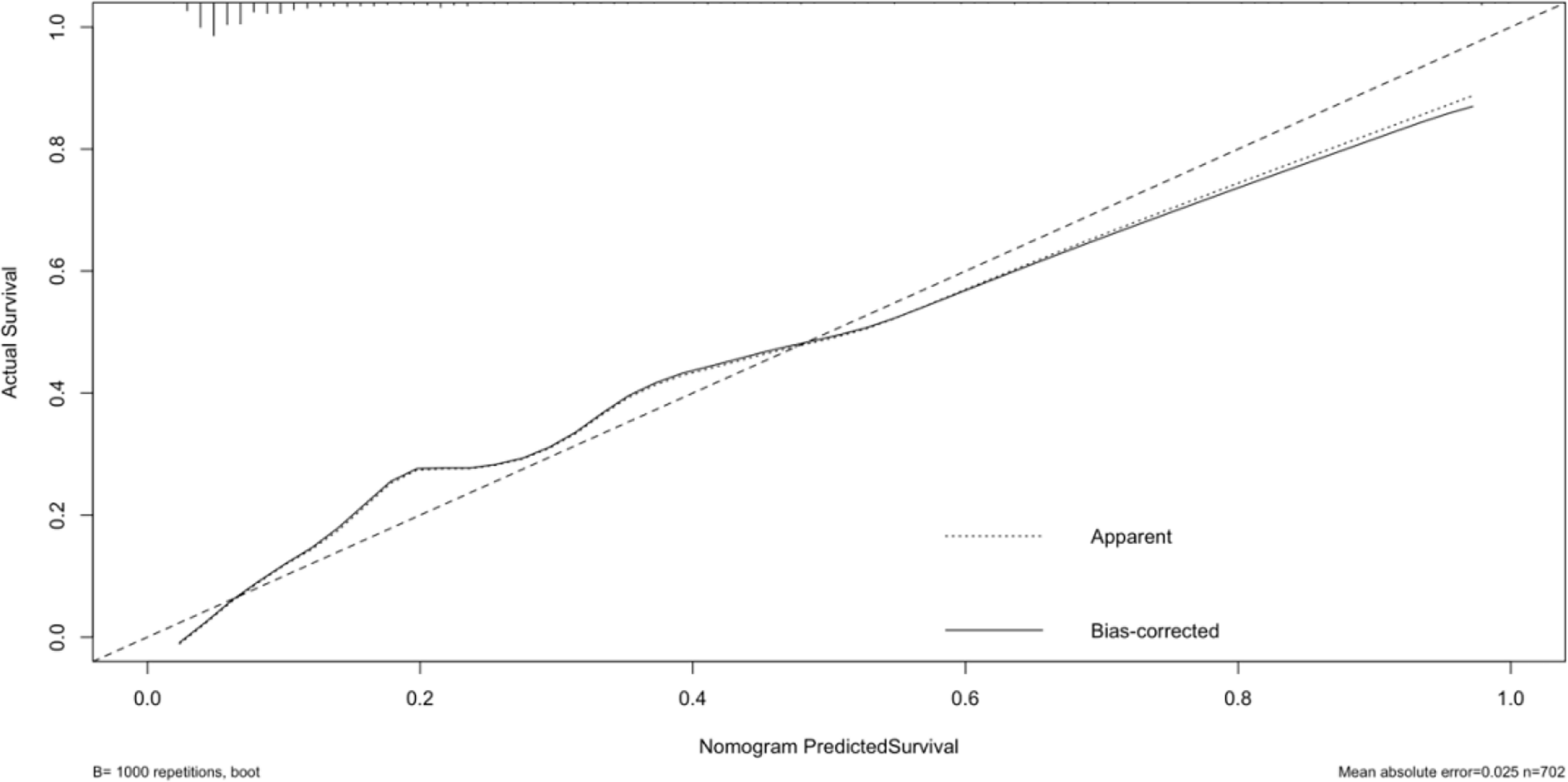
Calibration curve.

**Figure 4:**
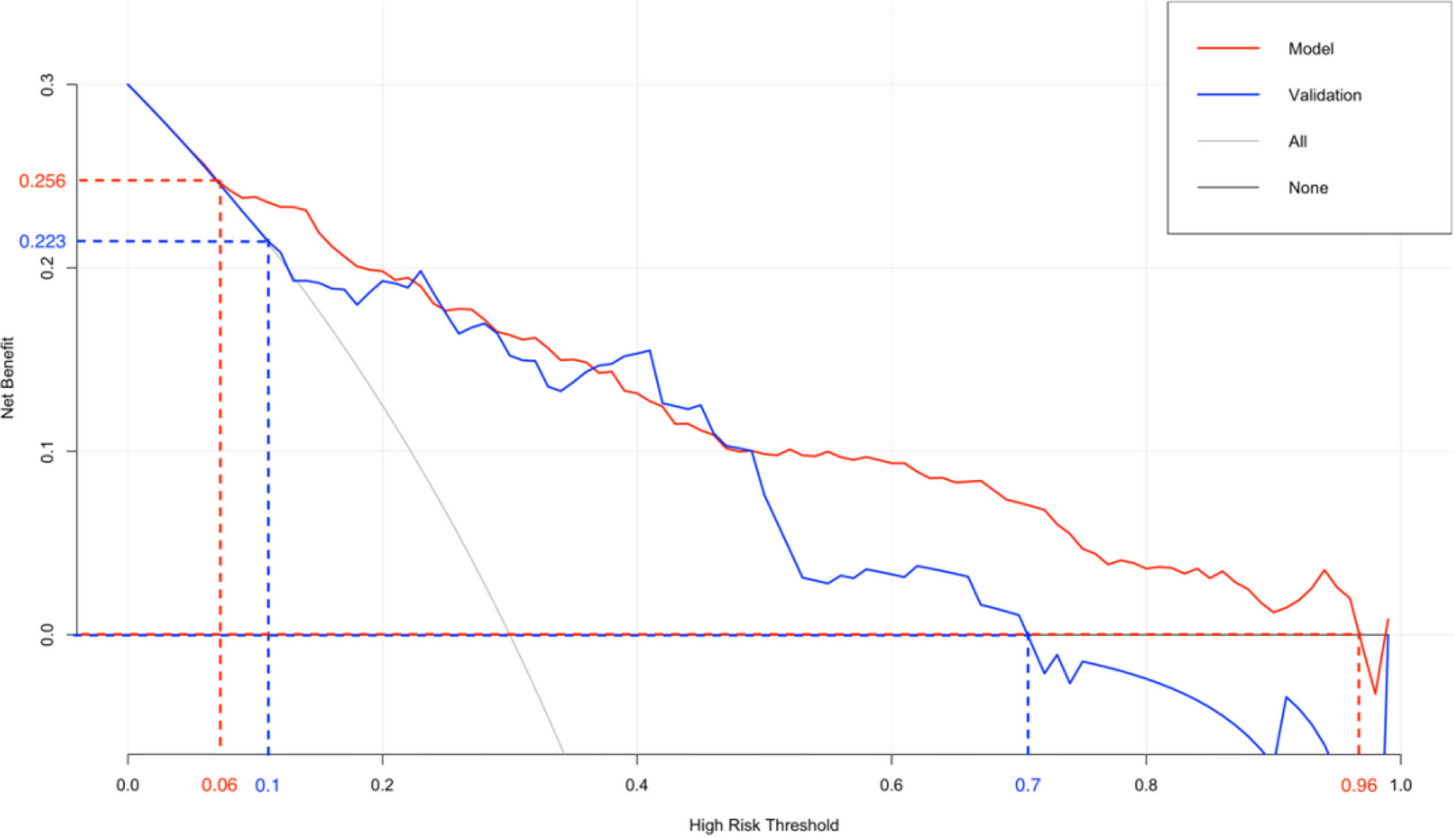
Decision curve analysis.

**Figure 5:**
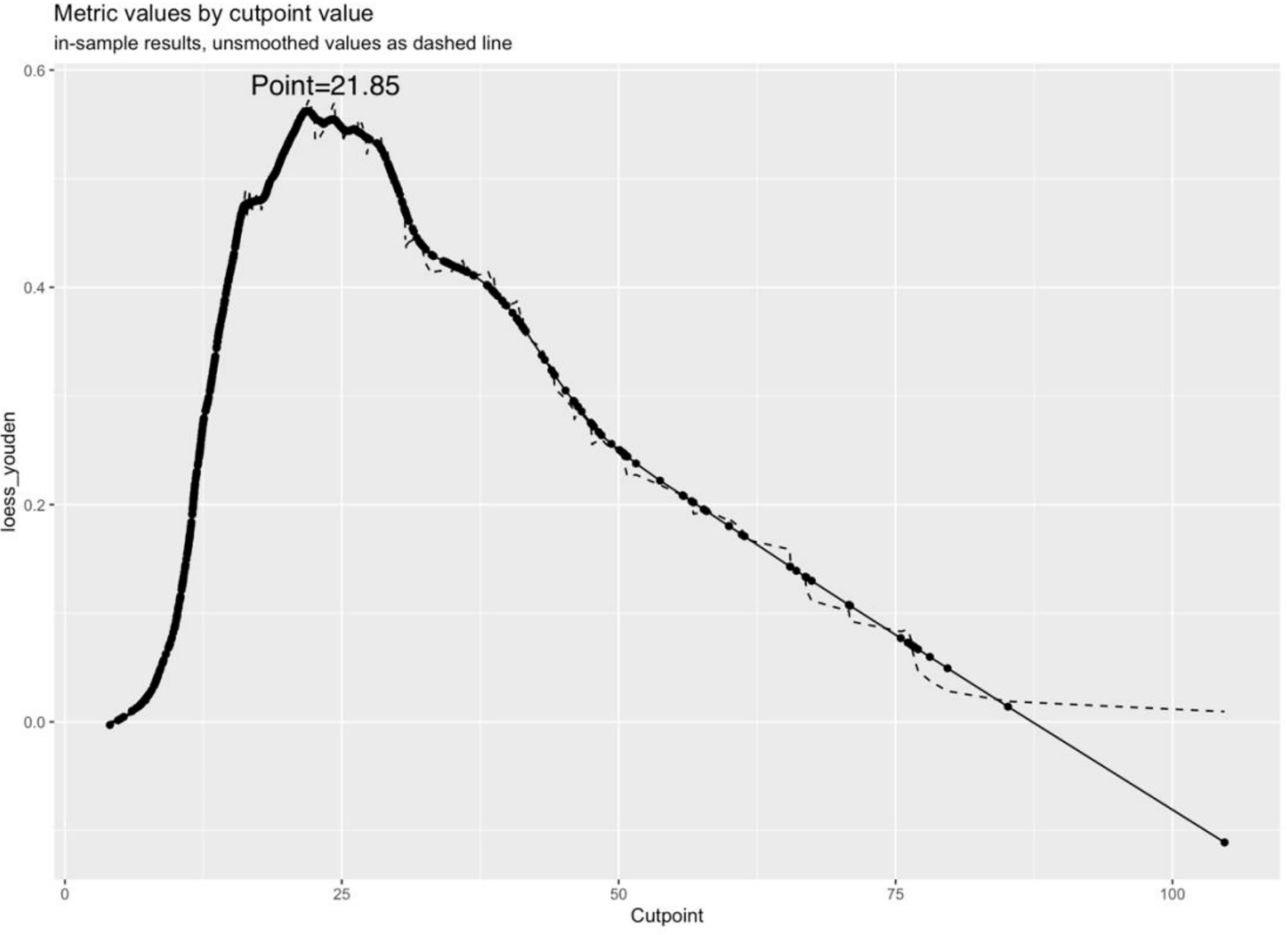
Youden curve and cut-off point.

The original dataset was re-sampled 1000 times by the bootstrap method to create a simulated dataset. The calibration curve showed that the predictive model had good agreement between the predicted discrimination rate and the actual discrimination rate of LLNM, with a mean absolute error of 0.025 (**Figure 3**). The Hosmer–Lemeshow test showed good goodness-of-fit (P = 0.448), indicating that the predictive model had good calibration ability. By DCA, no patient required further intervention, that is, no patient underwent lateral neck lymph node dissection, and the net benefit was 0. All represented clinical intervention, that is, all patients underwent lateral neck lymph node dissection, and the net benefit was the reverse of the backslash. When the predictive model curve was higher than the All, the predictive model had a net clinical benefit. **Figure 4** shows that in the net benefit range of 0–0.3, when the model risk threshold was in the range of 6–96%, the benefit of the intervention was significantly higher than that of no intervention or complete intervention, suggesting that the model has certain clinical utility (**Figure 4**). The Youden index of the nomogram was also calculated, and the cut-off point of the nomogram to predict LLNM in PTC was 21.85 (**Figure 5**).

#### Model external inspection

**Figure 6:**
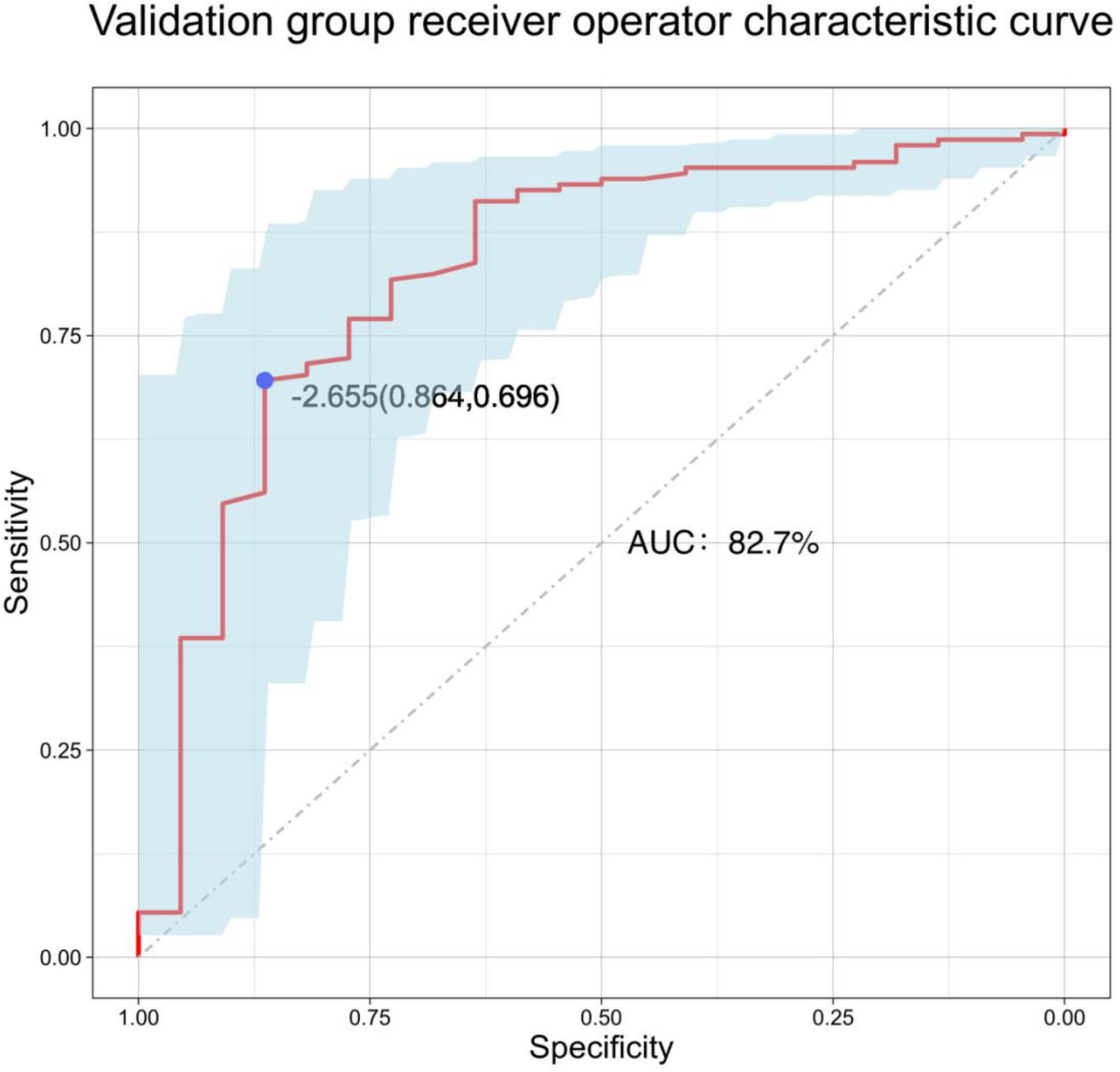
Validation group receiver operator characteristic curve.

A total of 171 external validation data were included to fit the model. The results showed that the AUC of the validation group was 0.827, and the 95% CI was 0.724–0.929 (P < 0.001). When the Youden index was at its highest, the specificity was 86.4% and the sensitivity was 69.6% (**Figure 6**). The Hosmer–Lemeshow test showed that the external validation data test model had better calibration (P = 1). By DCA, within a net benefit range of 0–0.3, when the model risk threshold was in the range of 10–70%, this predictive model yielded better clinical benefits (**Figure 4**).

## Discussion

Several studies have reported that PTC patients with LLNM have a higher risk of local recurrence and a poorer prognosis than those without LLNM (7, 8). The American Thyroid Association in 2015 (9) and the Chinese Society of Clinical Oncology in 2021 recommended dissection in cases of definite LLNM, that is, therapeutic lymph node dissection. For PTC patients without LLNM, lateral neck lymph node dissection does not reduce the risk of recurrence. In addition, it increases the risk of postoperative complications such as lymphatic leakage, damage to accessory nerves, and impaired neck muscle function. Presently, the preoperative assessment of the presence or absence of LLNM and the specific classification of metastatic lymph nodes are mainly based on imaging studies such as ultrasonography and computed tomography (CT). However, the diagnostic performance of both methods is limited because they often fail to detect metastatic lymph nodes, thus affecting the prognosis of patients (10). Therefore, a more convenient and efficient method was examined to assess LLNM in PTC patients before surgery and to formulate an appropriate method of cervical lymph node dissection. On the premise of ensuring the survival of patients, reducing the risk of postoperative complications, as well as recurrence, is critical in the diagnosis and treatment of PTC.

In recent years, many studies have demonstrated that immune function, inflammation, and tumor occurrence are closely related. For instance, immune-related factors can affect the tumor microenvironment and promote tumorigenesis (11, 12). Based on preoperative blood values detected by standard laboratory tests, preoperative immune indicators, including LMR, PLR, and NLR, are used because they can predict the presence of malignant tumors, such as lung cancer and prostate cancer, and predict the survival of patients (13, 14). In this study, several preoperative blood immune indexes, including LMR, PLR, NLR, and SII, reflected the immune function of PTC patients. Ceylan et al. studied the significance of NLR and PLR on PTC and showed that both parameters were associated with the clinicopathological invasiveness of PTC, and both could be used as markers for the risk assessment of PTC patients (5). However, in this study, LMR, PLR, and NLR had no significant effect on LLNM in PTC.

The absolute value of platelets × absolute value of neutrophils/absolute value of lymphocytes is a comprehensive index of platelets, neutrophils, and lymphocytes, and it can reflect immune function to a certain extent. Zheng et al. analyzed different indicators of 2415 patients with malignant tumors and reported that a high SII was a predictor of a worse prognosis of patients with malignancies (15). In a study of PTC patients, Muzza et al. reported the elevated expression of inflammatory molecules (16), while Zhang et al. studied 406 patients and showed that SII was a marker for predicting central lymph node metastasis (17). A high SII may indicate an impaired immune response, thus affecting the biological behavior of PTC. However, there is no study using SII to predict LLNM in PTC patients. This study is the first to find that a high SII is of great significance in PTC patients with LLNM. Combined with the results of previous studies, this may be related to the following points: (1) PTC patients show increased platelet counts. Platelets can induce endothelial cell proliferation, while increased levels of platelet factor and platelet endothelial growth factor can stimulate tumor cell proliferation and differentiation. In a study of thyroid diseases, Martin et al. reported that preoperative platelet counts were higher in patients with PTC than in those with benign thyroid lesions (18). Moreover, studies by Zhang et al. and Adewuyi et al. demonstrated that platelet-derived growth factor receptor α could up-regulate the mitogen-activated protein kinase/extracellular regulated protein kinase pathway, which promotes lymph node metastasis and invasion in PTC patients (19, 20). In other words, thrombocytosis is associated with the invasive ability of PTC. (2) PTC patients show increased neutrophil counts. Increased neutrophil counts associate with the release of several inflammatory mediators, which promote tumor development and changes in the tumor microenvironment. He et al. performed RNA sequencing and methylated RNA immunoprecipitation sequencing on PTC tissues and demonstrated that a reduction in the expression of METTL3, an RNA methyltransferase, can induce the accumulation of interleukin 8 and the recruitment of tumor-associated neutrophils, thus affecting the growth of papillary thyroid tumors (21), while Kucuk et al. reported that PTC with neutrophil infiltration was more aggressive, associating with a shorter disease-free survival time (22). In other words, with the increase in the number of neutrophils, the invasiveness of PTC increases. (3) PTC patients show decreased lymphocyte counts. In some solid tumors, as the number of lymphocytes decreases, the body’s ability to fight tumors also decreases (23). Pu et al. performed single-cell sequencing on PTC cells and demonstrated that CD8^+^ T cells, natural killer cells, and other lymphocytes interacted with tumor cells, indicating that immune regulation plays important roles in the cellular ecology and development of PTC (4). Zhu et al. confirmed that PTC was accompanied by marked T lymphocyte depletion, in which the number of lymphocytes in the blood of PTC patients was reduced (24). Therefore, a high SII has a certain relationship with the increased invasive ability of PTC, which can be used as a marker to predict LLNM in PTC patients.

Feng et al. studied the predictors of lymph node metastasis in the central and lateral cervical regions and observed that tumor T stage, that is, tumor diameter, was an independent risk factor of LLNM that affected the recurrence-free survival of PTC patients (25). Several studies have reported that the larger the tumor diameter, the higher the risk of LLNM in PTC (26, 27), which is consistent with the results of this study. These findings suggest that PTC patients with a larger tumor diameter should undergo rigorous preoperative examinations, together with an evaluation of the status of LLNM to avoid misdiagnosis and reduce the risk of local recurrence after surgery.

The nomogram is based on logistic multivariate regression analysis, and it integrates the influencing factors and converts the regression coefficients into line segments in proportion to the graph. Scores are assigned according to the value of each influencing factor, and the total scores are determined to obtain the predicted value of the individual events through a conversion relationship between the total score and the outcome probability. In this study, for the first time, preoperative blood immune indexes were used to predict LLNM in PTC, and it was confirmed that tumor diameter and SII were independent risk factors for LLNM in PTC. At the same time, a predictive model for assessing LLNM in PTC patients was constructed and verified. The AUC of the predictive model was 0.860, and a nomogram was constructed (**Figure 1**). The external validation data of our research center fit the model. The AUC was 0.827, and the model prediction effect was good. The specificity (80.2% vs 86.4%) and sensitivity (77.0% vs 69.6%) were also higher in the model and external validation groups. The bootstrap method (**Figure 3**) indicated that the calibration curve was close to the ideal curve, indicating that the model had a good predictive ability. The Hosmer–Lemeshow test confirmed the internal and external verification, and indicated that the predictive model had good discrimination and calibration. In recent years, several investigators have used DCA to evaluate the clinical benefit rate. This study shows that in the model group, with a risk threshold of 6–96%, the predictive model showed a significant clinical benefit, and in the external validation group, with a risk threshold of 10–70%, there was also a certain clinical benefit (**Figure 4**). In summary, this predictive model can better predict LLNM in PTC patients, and it is convenient for use in clinical settings, with high accuracy and repeatability. It can be used as a preoperative assessment method for LLNM in PTC patients to formulate individualized cervical lymph node dissection protocols.

The shortcomings of this study are as follows: (1) The newly established predictive model consisted of a single-center study, and subsequent studies should include more centers, involve large-sample verification, and improve the accuracy of the predictive model. (2) Several preoperative blood indicators were not examined, and follow-up studies should incorporate additional indicators to improve the predictive accuracy of the model. (3) This study predicted LLNM in PTC patients but did not examine the specific lateral neck regions. In the future, a prediction method for specific classifications should be devised.

## Data availability statement

The raw data supporting the conclusions of this article will be made available by the authors, without undue reservation.

## Ethics statement

The studies involving human participants were reviewed and approved by the Ethics Committee of Affiliated Hangzhou First People’s Hospital Zhejiang University School of Medicine. Written informed consent to participate in this study was provided by the participants’ legal guardian/next of kin.

## Author contributions

DL had full access to all of the data in the manuscript and takes responsibility for the integrity of the data and the accuracy of the data analysis. All authors read and approved the final manuscript. Concept and design: all authors. Acquisition, analysis, or interpretation of data: all authors. Drafting of the manuscript: LZ and TZ. Critical revision of the manuscript for important intellectual content: LZ, TZ, and DL. Statistical analysis: LZ and TZ. Supervision: YZ and DL.

## Funding

This work was supported by the Project of Medical Scientific and Technology Program in Hangzhou (grant number: A20200432), and Zhejiang Province Public Welfare Technology Application Research Project (grant number: GF22H165705).

## Conflict of interest

The authors declare that the research was conducted in the absence of any commercial or financial relationships that could be construed as a potential conflict of interest.

## Publisher’s note

All claims expressed in this article are solely those of the authors and do not necessarily represent those of their affiliated organizations, or those of the publisher, the editors and the reviewers. Any product that may be evaluated in this article, or claim that may be made by its manufacturer, is not guaranteed or endorsed by the publisher.
